# Vestibular schwannoma: genetic and epigenetic mechanisms, hearing loss, and emerging therapies

**DOI:** 10.1007/s11060-026-05621-4

**Published:** 2026-05-16

**Authors:** Franciska Otaner, Vratko Himic, Luis O. Vargas, Matthew Abikenari, Neelesh Pandey, Shayndhan Sivanathan, Olivia Kalmanson, Aparna Govindan, Diane Jung, Dagoberto Estevez-Ordonez, Amy Wang, Sanjeeva Jeyaretna, Ashish H. Shah, Ricardo J. Komotar, Bradley Gampel, Christine Dinh, Michael E. Ivan

**Affiliations:** 1 Faculty of Medicine and Health Sciences, McGill University, Montreal, QC Canada; 2 Department of Neurological Surgery, University of Miami Miller School of Medicine, Miami, FL USA; 3 Department of Neurosurgery, University at Buffalo Jacobs School of Medicine and Biomedical Sciences, Buffalo, NY USA; 4 Department of Neurosurgery, Stanford University School of Medicine, Stanford, CA USA; 5 Yale College, New Haven, Connecticut, USA; 6 Department of Neurosurgery, University of Oxford, Oxford, UK; 7 Department of Otorhinolaryngology, University of Miami Miller School of Medicine, Miami, FL USA; 8 Department of Neuro-Oncology, University of Miami Miller School of Medicine, Miami, FL USA

**Keywords:** Vestibular schwannoma, Acoustic neuroma, Neurofibromatosis, NF2, Schwannomatosis, Epigenetics

## Abstract

**Background:**

Although most vestibular schwannomas (VS) occur sporadically, both sporadic and hereditary tumors share common molecular features beyond the loss of *NF2*. New evidence highlights the role of interconnected signaling pathways and epigenetic regulation in Schwann cell tumorigenesis, pointing toward potential molecularly targeted therapeutic strategies.

**Methods:**

This review synthesizes preclinical, molecular, and clinical evidence to examine genetic and epigenetic mechanisms underlying VS, therapeutic strategies, and contributors to hearing loss. A structured search of ClinicalTrials.gov identified 21 Phase 1–3 interventional therapeutic trials.

**Results:**

VS pathogenesis is driven by *NF2* loss and merlin deficiency, leading to dysregulation of Hippo/YAP-TAZ, PI3K/AKT/mTOR, VEGF, MAPK, and adhesion pathways. Epigenetic alterations, including DNA methylation, chromatin remodeling, non-coding RNAs, and SOX10 dysfunction, further shape tumor behavior. Clinical trial analysis revealed a predominance of early-phase, non-comparative studies, limited progression to later-phase trials, and incomplete results reporting, indicating gaps in high-quality evidence. Bevacizumab remains the most consistent systemic therapy for select *NF2*-related cases, while other agents such as icotinib, lapatinib, everolimus, selumetinib, and brigatinib have shown modest activity, primarily disease stabilization. Emerging approaches, including TEAD inhibition, PI3K/mTOR blockade, MEK inhibition, and combined signaling-epigenetic strategies, show preclinical promise. Hearing loss is multifactorial, involving tumor-secreted factors, inflammation, vascular changes, and inner ear damage alongside nerve compression.

**Conclusion:**

VS biology reflects integrated genetic and epigenomic dysregulation. Advancing care will require multi-omic classification, biomarker-driven trials, and combination therapies targeting both signaling and epigenetic vulnerabilities. Future management is expected to shift toward personalized, mechanism-based strategies aimed at durable tumor control while preserving hearing and quality of life.

**Supplementary Information:**

The online version contains supplementary material available at 10.1007/s11060-026-05621-4.

## Introduction

Vestibular schwannomas (VS) are benign Schwann cell tumors of the vestibulocochlear nerve, accounting for ~ 5–6% of intracranial tumors and many cerebellopontine angle masses [1]. Despite slow growth, they can cause progressive unilateral hearing loss, tinnitus, imbalance, and cranial nerve deficits, with larger tumors leading to brainstem compression, hydrocephalus, and neurological decline [2]. Management includes observation, microsurgery, and stereotactic radiosurgery, guided by tumor characteristics, hearing status, comorbidities, and patient preference [3]. Given the need to balance tumor control with functional preservation, multidisciplinary care is essential.

Approximately 90% of VS are sporadic, and the remaining genetically associated cases are limited to *NF2*-related schwannomatosis (NF2-SWN), LZTR1-related schwannomatosis (LZTR1-SWN), and Carney complex caused by *PRKAR1A* mutations. Despite being non-hereditary, most sporadic VS also show somatic *NF2* loss, with one study reporting NF2 alterations in over 95% of cases [4]. Other schwannomatoses (e.g., *SMARCB1*, 22q, *DGCR8*) are typically not linked to VS [5]. Although sporadic unilateral VS typically presents later in adulthood, with a median age at diagnosis of approximately 60 years, individuals with NF2-SWN are usually diagnosed much earlier, most commonly between 18 and 24 years of age [2, 6].

Loss of merlin activates multiple interconnected pathways, including Hippo, PI3K/AKT/mTOR, VEGF-mediated angiogenesis, MAPK, and adhesion/motility signaling such as FAK, c-MET, and EphA2 [7–9]. These pathways drive Schwann cell proliferation, survival, and microenvironment remodeling. Additional alterations, including mutations in other 22q tumor suppressors like *LZTR1* and mosaic *NF2* mutations, further influence penetrance, growth, and clinical features [10]. Beyond NF2 loss and canonical signaling, epigenetic regulation also plays a key role in VS biology through developmental hypomethylation patterns, methylation-defined subgroups, chromatin remodeling, non-coding RNAs, and *SOX10* [11–15]. These findings indicate that VS behavior reflects combined genetic and epigenomic dysregulation.

Therapeutic translation has been inconsistent. Multiple agents have been tested in *NF2*-related and sporadic VS, but most have shown only modest or variable effects on tumor control and hearing outcomes. Among currently available systemic therapies, bevacizumab remains the most effective option for select NF2-SWN tumors [16]. Moreover, integrated molecular diagnostics are now standard for many CNS tumors; in tumors such as pediatric high-grade gliomas and diffuse midline gliomas, combined genomic and epigenomic profiling has improved classification and helped guide therapy. Similar approaches may also support prognosis, treatment selection, and clinical monitoring in VS [17]. Despite expanding molecular insight and a growing number of investigational therapies, progress in translating these advances into consistent clinical benefit remains limited, and key gaps persist in integrating genetic and epigenetic knowledge into patient stratification and treatment design. As multi-omic technologies and targeted therapeutic strategies continue to evolve, there is a need for a clear and clinically relevant synthesis to bridge these gaps and guide future research and management.

To address this, we provide a comprehensive narrative review of the genetic and epigenetic pathways underlying VS. We also evaluate completed and ongoing clinical trials targeting genetically-driven molecular mechanisms and examine emerging epigenetic regulation. Finally, we explore how these processes may contribute to disease phenotypes, particularly hearing loss, a key determinant of patient quality of life.

## Methods

### Study design

This study was conducted as a hybrid narrative and systematic review, integrating a comprehensive systematic search and predefined study selection process with a structured, hypothesis-generating narrative synthesis to characterize the genetic, epigenetic, and translational landscape of VS. This approach was chosen to allow rigorous identification and selection of relevant studies while enabling integration of heterogeneous evidence spanning preclinical, molecular, and clinical domains. Methodological conduct and reporting were guided by the principles of the PRISMA framework where applicable.

### Systematic review

To characterize the current translational and investigational therapeutic landscape of VS, a systematic search of ClinicalTrials.gov was performed using the terms “vestibular schwannoma” and “acoustic neuroma,” with the final search conducted on November 30, 2025. Studies were eligible if they were Phase 1 through Phase 3 prospective interventional trials evaluating therapeutic strategies in VS. Early Phase 1 pharmacokinetic-only studies, Phase 4 trials, and observational studies were excluded to maintain a more methodologically consistent synthesis of prospective interventional trials focused on whether candidate therapies demonstrate clinical efficacy in VS, including tumor control, hearing outcomes, or radiographic response. This approach avoided combining efficacy-focused trial data with pharmacokinetic studies assessing drug exposure and delivery, as well as post-marketing and observational studies primarily reflecting long-term safety, durability, and real-world treatment experience.

Study identification and selection were conducted independently by two reviewers (F.O. and N.P.), with discrepancies resolved by a third reviewer (L.V.). Data were extracted using a predefined framework. Evidence assessment was also performed independently by the same reviewers using a structured qualitative approach.

This process yielded 113 clinical trials, of which 21 met inclusion criteria and were included in the qualitative synthesis. Data extracted from included trials included study title, trial identifier, study start date, study status, study design and summary, and reported outcomes or available results from ClinicalTrials.gov and, when available, corresponding published studies; these data were subsequently synthesized qualitatively.

Evidence strength was evaluated based on study phase, design, sample size, and reported clinical outcomes, including radiographic response, tumor control, and hearing preservation. Greater weight was assigned to later-phase studies and those demonstrating consistent findings across cohorts, while early-phase trials were interpreted cautiously given their exploratory nature and limited sample sizes.

### Narrative synthesis

A comprehensive literature search was performed using PubMed from database inception through November 30, 2025. As this was a narrative review, studies were not screened or selected according to a predefined systematic review protocol. Instead, article selection and synthesis were guided by relevance to the review objectives and by the multidisciplinary expertise of the co-authors in neurosurgery, neuro-oncology, and otolaryngology. This search was designed to support a structured narrative synthesis of the genetic, epigenetic, and molecular mechanisms underlying VS using controlled vocabulary and free-text terms. Reference lists of relevant articles were also screened to identify additional studies. Studies were selected based on relevance to the scope of this review, with emphasis on mechanistic insight, translational relevance, and clinical applicability. Findings were synthesized qualitatively across key domains, including genetic drivers and epigenetic regulation, and were further organized to examine underlying molecular mechanisms, therapeutic strategies, preclinical and clinical evidence, and their impact on symptomatology. Strength of evidence was interpreted based on biological plausibility (i.e., consistency with established molecular mechanisms), consistency across experimental models, and relevance to human disease. Preclinical findings were considered hypothesis-generating and were integrated with available clinical evidence where possible to support translational interpretation. Study selection, synthesis, and interpretation were performed collaboratively by the authors.

## Genetically-driven molecular pathways of vestibular schwannomas

### NF2-SWN as a model

NF2-SWN is often studied to understand sporadic VS, as both share *NF2* loss and overlapping molecular programs despite clinical differences. The *NF2* gene on chromosome 22 encodes merlin (neurofibromin 2), a regulator of cell growth; biallelic loss promotes tumor formation [6].

Inflammatory signaling follows a similar trajectory, particularly pathways related to macrophage activity, angiogenesis, and inflammation. Bulk transcriptomic and imaging mass cytometry studies show that macrophages are the dominant immune population in VS, comprising about one-third of tumor mass in both NF2-SWN and sporadic cases, along with regulatory T cells present at lower levels. Notably, no significant differences have been observed in signaling pathways, gene expression, cell-type composition, or cytometry profiles between the two groups, indicating a highly similar tumor immune microenvironment [24]. Overall, sporadic and hereditary schwannomas share similar pathways, making hereditary forms useful models for studying the more common sporadic cases [10]. Thus, NF2-SWN provides a useful model due to its more abundant and predictable tumors, which reflect pathways active in sporadic VS. However, this approach should be applied cautiously, and it is crucial to emphasize that sporadic VS shows additional molecular and epigenetic heterogeneity beyond features captured in NF2-SWN, underscoring the need for broader investigation [25]. For example, the recurrent somatic *SH3PXD2A-HTRA1* gene fusion, resulting from a balanced chromosomal inversion on 10q, has been identified in approximately 10% of sporadic schwannomas and drives tumorigenesis through MEK-ERK pathway activation independent of *NF2* loss [26].

### Two-hit hypothesis

NF2-SWN is an autosomal dominant disorder caused by mutations in the *NF2* gene on chromosome 22q; ~50% of cases are inherited, while the remainder arise de novo, with 25–50% showing somatic mosaicism [6]. VS pathogenesis follows the classic two-hit model, requiring biallelic *NF2* inactivation via germline or somatic mutation and a second event such as loss of heterozygosity, uniparental disomy, or promoter methylation [27].

Some tumors appear monoallelic due to detection limits. Undetected structural variants, deep intronic mutations, or low-level mosaicism may obscure a second hit. Deep sequencing has identified post-zygotic *NF2* mutations in such cases, often associated with later onset and milder disease, underscoring the importance of molecular testing in atypical or unilateral presentations [28].

**Fig. 1:**
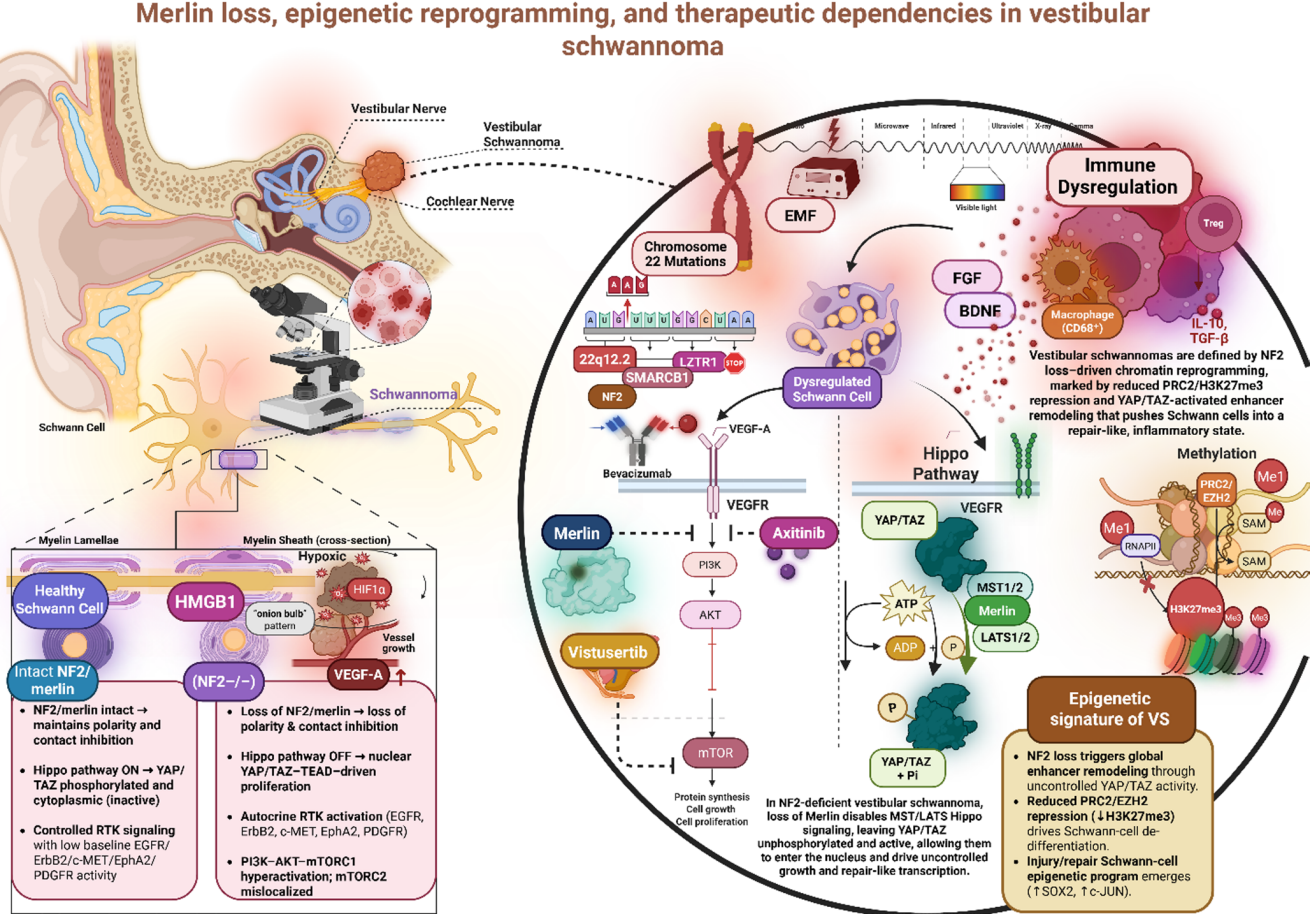
Integrated molecular, epigenetic, and therapeutic landscape of vestibular schwannoma (VS). Loss of NF2/Merlin disrupts Schwann cell polarity and contact inhibition, disables Hippo signaling (MST1/2-LATS1/2), and permits YAP/TAZ-driven transcription of proliferative, extracellular matrix remodeling, and repair phenotype programs. NF2 loss also amplifies receptor tyrosine kinase signaling (EGFR, ErbB2, MET, PDGFRA) and hyperactivates the PI3K–AKT–mTOR axis, creating targetable dependencies (e.g., vistusertib, VEGFR inhibitors). Epigenetic reprogramming includes PRC2/EZH2-mediated H3K27me3 repression, enhancer activation at YAP/TAZ-bound loci, and SOX2/c-JUN-driven repair-like chromatin states. Hypoxia and HIF1α stabilization drive VEGF-A production and vascular dysregulation, while the tumor immune microenvironment is enriched for CD68 + macrophages and regulatory T cells producing IL-10 and TGF-β. Together, these alterations define the pathogenic architecture of VS and highlight key nodes for therapeutic intervention

Promoter methylation or transcriptional repression may act as a functional second hit, while true *NF2* haploinsufficiency is rare but may occur with loss of other 22q tumor suppressors such as *LZTR1*. In one series, a sporadic VS showed biallelic *LZTR1* loss with monoallelic *NF2* loss, though mutation type did not correlate with clinical outcomes [29]. *LZTR1* encodes a CUL3 adaptor that regulates RAS degradation; its loss leads to RAS/MAPK activation independent of merlin [30]. Unilateral VS may occur in *LZTR1*-related schwannomatosis, but bilateral VS remains diagnostic of NF2-SWN [31] (Fig. 1).

### The hippo pathway

*NF2* encodes merlin, a membrane-cytoskeleton scaffold that regulates cell architecture, adhesion, and growth [7]. In Schwann cells, merlin maintains polarity and activates Hippo signaling, enabling LATS1/2 to keep YAP/TAZ phosphorylated and cytoplasmic, enforcing quiescence [32]. Loss of merlin disrupts polarity, impairs Hippo signaling, and allows nuclear YAP/TAZ-TEAD-mediated transcription of proliferative and pro-survival genes. Merlin-deficient cells also produce ErbB ligands, activating EGFR and downstream Src and PI3K/Akt signaling via autocrine loops, contributing to heterogeneity in proliferative states [32, 33].

Human VS show reduced phospho-LATS/YAP and increased nuclear YAP, correlating with proliferation [34]. Genetic models confirm that loss of *LATS1/2* induces schwannomas, while deletion of *YAP/TAZ* prevents tumor formation [33]. Restoration of merlin or Hippo signaling reestablishes YAP cytoplasmic sequestration and growth control [32]. Preclinical studies identify the YAP/TAZ-TEAD complex as crucial for proliferation and survival of VS cells, with TEAD palmitoylation inhibitors (e.g., VT1, VT2) suppressing tumor growth, though clinical safety and efficacy remain unestablished [35]. No Hippo pathway-directed agent has yet entered clinical trials specifically for VS.

### The PI3K/Akt/mTOR axis

The PI3K/AKT/mTOR pathway regulates growth, metabolism, and survival and is consistently upregulated in VS [36]. Normally, receptor tyrosine kinase (RTK) signaling activates PI3K/AKT and downstream mTOR complexes: mTORC1 drives protein synthesis and proliferation, while mTORC2 supports cytoskeletal organization and cell polarity [37]. Merlin restrains mTORC1 and preserves mTORC2 function; its loss causes chronic mTORC1 activation, disordered mTORC2 signaling, persistent AKT activation, and loss of polarity. mTORC1 inhibition can also relieve negative feedback, paradoxically reactivating PI3K/AKT, underscoring the need for multi-targeted approaches [37]. Thus, in merlin-deficient cells, feedback regulation is disrupted, and targeting a single pathway node may unintentionally enhance signaling, highlighting the need for multi-targeted therapeutic strategies.

Preclinical studies show that dual PI3K/mTOR inhibitors (e.g., BEZ235) reduce schwannoma cell viability [36]. In contrast, clinical trials of everolimus (mTORC1 inhibitor) in NF2-SWN have shown limited benefit, with slowed growth in some cases but no consistent radiographic or hearing improvement in Phase II studies [20]. These findings suggest that broader pathway inhibition may be required, though this remains unproven clinically.

### Angiogenesis and the VEGF pathway

VS are highly angiogenesis-dependent, driven by VEGF-A. Merlin loss shifts the balance toward VEGF signaling by reducing anti-angiogenic factors (e.g., semaphorins), producing leaky, disorganized vasculature that contributes to edema and hypoxia [38]. Bevacizumab, an anti-VEGF-A antibody, is the most consistently effective systemic therapy for NF2-SWN and is the NCCN-preferred treatment option. In some patients, it improves hearing and controls tumor growth, potentially through vascular normalization, reduced edema, and improved tissue oxygenation [39]. Pooled data show radiographic responses in 38% and hearing responses in 45%, with some patients improving clinically before tumor shrinkage [40, 41]. Responses appear age-dependent, with younger patients showing higher rates of radiographic progression, and biomarker analyses suggest that baseline tumor vessel permeability and plasma hepatocyte growth factor levels may predict imaging and hearing responses, respectively [42, 43]. In addition to variability in patient response, bevacizumab often requires continuous therapy and is limited by treatment-related toxicities, including hypertension, proteinuria, and fatigue, as well as tumor regrowth after discontinuation [16, 44].

### The Ras/Raf/MEK/ERK (MAPK) pathway

The Ras/Raf/MEK/ERK (MAPK) pathway drives proliferation and survival downstream of receptor tyrosine kinases (e.g., ErbB, c-MET). Merlin normally restrains this signaling by limiting Ras activation and RTK clustering; its loss leads to constitutive Ras/ERK activation and growth independent of external signals. Elevated phospho-ERK is observed in both *NF2-*related and sporadic VS, highlighting MAPK as a key effector and therapeutic target [8]. Preclinical studies show that MEK inhibitors (e.g., trametinib, PD0325901, cobimetinib) significantly reduce proliferation in *NF2*-deficient Schwann cells and VS models [45].

### FAK, c-MET, and EphA2 signaling (adhesion and motility axis)

In normal Schwann cells, merlin stabilizes focal adhesions and restrains FAK activation. Its loss leads to constitutive FAK signaling, cytoskeletal remodeling, and increased survival and invasiveness. This involves overexpression of c-MET and EphA2, which activate PI3K/AKT and MAPK pathways to promote growth and migration. Targeted therapies show promise: crizotinib (c-MET, indirect FAK inhibition) has demonstrated preclinical efficacy, while brigatinib (FAK1, EphA2) has shown clinical activity. Neratinib, a pan-HER inhibitor, is also under investigation in NF2-SWN [9].

### Genotype-phenotype correlations in NF2-SWN

Genotype-phenotype correlations are well established in NF2-SWN. Truncating mutations are associated with more severe disease, including younger age of symptom onset, higher tumor burden, and faster VS growth rates, whereas missense mutations are associated with milder phenotypes [18]. The UK NF2 Genetic Severity Score formalizes this relationship, with higher scores predicting worse morbidity and mortality [46].

## Clinical translation of genetically mediated molecular pathways

### Characteristics of investigational therapeutic clinical trials

**Fig. 2:**
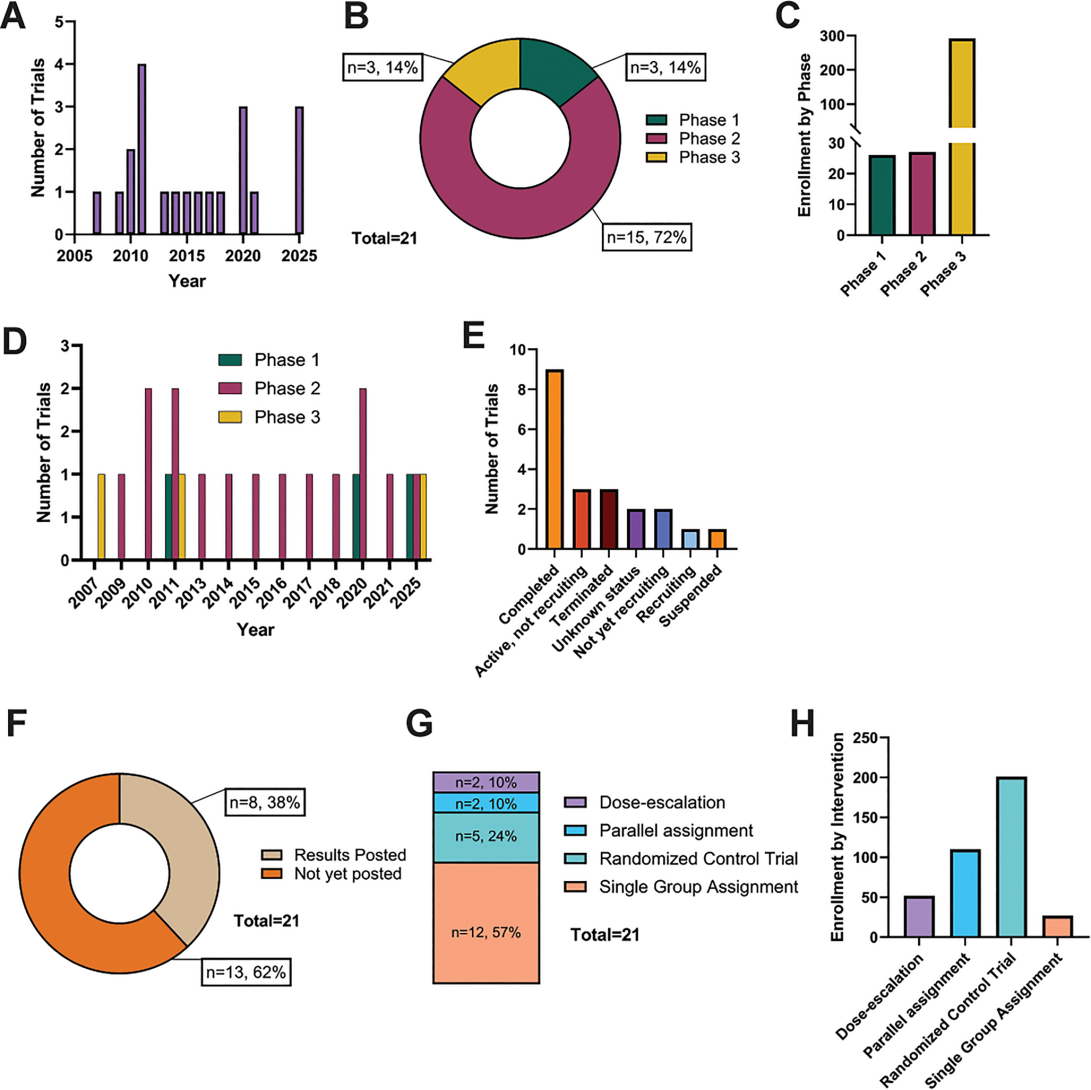
Descriptive summary results of systematic synthesis of interventional therapeutic clinical trials in VS. **A**: Breakdown of trials between 2005 and 2025. **B**: Breakdown of trial numbers by Phase 1–3. **C**: Mean enrolled number of patients across all trials stratified by Phase. **D**: Breakdown of number of trials by phase across the study period. **E**: Breakdown of number of trials by study status. **F**: Pie chart of number and percentage of trials that have successfully posted results and those that have not. **G**: Breakdown of trial type by interventional model (dose-escalation, parallel assignment, randomized control trial or single group assignment). **H**: Mean enrolled number of patients across all trials stratified by interventional model

A total of 21 clinical trials were included, most of which were Phase 2 (*n* = 15, 72%), with fewer Phase 1 (*n* = 3, 14%) and Phase 3 (*n* = 3, 14%) studies (Tables 1, 2 and 3 and see Supplementary File 1 for PRISMA flowchart of clinical trial identification, screening and selection). These trials evaluated therapies targeting genetically-driven molecular VS pathways, with no current clinical trials specifically targeting epigenetic mechanisms. The tables indicate whether each trial included children and whether the VS population was specified as sporadic or NF2-SWN, as well as patient-relevant outcomes and documented adverse effects. Trials spanned 2007–2025 (median start year 2015), with early clustering around 2010–2011 and renewed activity in 2020 and 2025 (Fig. 2a); however, this did not translate into progression to later-phase studies, suggesting stagnation in clinical advancement (Fig. 2b-d).

**Table 1:** Summary of phase 1 interventional clinical trials in vestibular schwannoma identified through ClinicalTrials.gov

Title	Trial ID	Study Start	Study Status	Enrollment	Interventional Model	Summary	Results
Super-Selective Intraarterial Cerebral Infusion of Bevacizumab (Avastin) for Treatment of Vestibular Schwannoma	NCT01083966	2011	Suspended	*N* = 30	Single Group Assignment	This study tested whether bevacizumab could be safely delivered through a single superselective intra-arterial infusion to patients with VS, aiming to increase drug delivery to the tumor while minimizing systemic effects. The primary outcome was determining the maximum tolerated dose. Secondary outcomes included evaluating the composite overall response rate, six-month progression-free survival, and hearing response in eligible patients.	No results posted.
Triamcinolone Levels in Cochlear Perilymph, Lateral Canal and CSF	NCT04658836	2020	Completed	*N* = 21	Single Group Assignment	This study enrolled patients undergoing VS surgery who received intratympanic triamcinolone acetonide 24 h before the procedure. Perilymph and cerebrospinal fluid were collected intraoperatively to measure drug levels. The study aimed to clarify how triamcinolone spreads from the middle ear into inner-ear fluids and whether it reaches the cerebrospinal fluid, with the primary outcome being the triamcinolone acetonide level in cochlear perilymph, lateral canal and CSF.	Triamcinolone was consistently detectable in perilymph at both sampling sites, showing similar concentrations, but was found in the cerebrospinal fluid in only 43% of patients and at very low levels. Perilymph and CSF concentrations did not correlate, and cochlear aqueduct width or age did not predict CSF penetration [18].
Anti-VEGF Gene Therapy Trial for Vestibular Schwannoma	NCT06517888	2025	Recruiting	*N* = 27	Dose-escalation, sequential assignment (non-randomized)	This trial will assess the safety and tolerability of a single unilateral dose of AAVAnc80-antiVEGF at three dose levels and evaluate whether the Akouos delivery device can reliably and safely deliver the gene therapy to VS, with the primary outcome being the frequency of adverse events (AEs). Secondary outcomes include tumor volume and performance of the Akouos delivery device.	No results posted.

**Table 2:** Summary of phase 2 interventional clinical trials in vestibular schwannoma identified through ClinicalTrials.gov

Title	Study ID	Study Start	Study Status	Enrollment	Interventional Model	Summary	Results
Lapatinib Study for Children and Adults With Neurofibromatosis Type 2 (NF2) and *NF2*-Related Tumors	NCT00973739	2009	Completed	*N* = 21	Single Group Assignment	This study evaluated the efficacy of lapatinib in children and adults with VS, based on its inhibition of ErbB2 and EGF signaling implicated in tumor growth. The primary outcome was estimated volumetric progression-free survival at 12 months. Secondary outcomes included hearing-related volumetric progression-free survival at 12 months and the frequency of Grade 1–2 and Grade 3 toxicities (CTCAE).	Estimated 12-month volumetric PFS was 70.6%, and hearing-related PFS was 88.9%. Grade 1–2 toxicities were common (21/21 patients), while Grade 3 toxicity was rare (1 patient) [19].
A Study of Nilotinib in Growing Vestibular Schwannoma	NCT01201538	2010	Terminated	*N* = 2	Single Group Assignment	This study evaluated the efficacy of Nilotinib for patients with progressing VS, based on its ability to inhibit RTK pathways known to be overactive in both sporadic and *NF2*-related tumors. The primary outcome was the change in VS volume, with secondary aims including assessment of toxicity, quality of life, and symptom management during treatment.	No results posted.
Bevacizumab for Symptomatic Vestibular Schwannoma in Neurofibromatosis Type 2 (NF2)	NCT01207687	2010	Completed	*N* = 14	Single Group Assignment	This study observed the activity, safety, and tolerability of intravenous bevacizumab given every three weeks in NF2 patients with VS, with the primary outcome being the proportion of patients who achieved a hearing response. Secondary measures included toxicity, radiographic response, changes in tumor volume and auditory function, vascular permeability, and multiple quality-of-life assessments.	About one-third of patients had a hearing response, and serious toxicities were uncommon. Around 40–50% showed a radiographic response or meaningful tumor-volume reduction. Auditory function measures were limited but generally stable, and vascular permeability data were too sparse for conclusions. Quality-of-life scores across multiple domains remained overall stable during treatment.
Hearing Outcomes Using Fractionated Proton Radiation Therapy for Vestibular Schwannoma	NCT01199978	2011	Active, not recruiting	*N* = 30	Single Group Assignment	This study examines fractionated proton radiotherapy, chosen for its ability to spare normal tissue, and its combination with losartan to assess potential benefits for patients with VS. The primary outcome is long-term hearing effects. Secondary outcomes include local tumor control, dosimetric parameters, second-tumor incidence, toxicity of losartan with proton therapy, and losartan’s impact on hearing preservation and circulating biomarkers.	No results posted.
Efficacy and Safety Study of RAD001 in the Growth of the Vestibular Schwannoma(s) in Neurofibromatosis 2 (NF2) Patients (AFINF2)	NCT01490476	2011	Completed	*N* = 10	Single Group Assignment with External Control	This study tested whether RAD001 (everolimus) could shrink or slow VS growth in NF2 patients. The primary outcome was its effect on VS growth measured by MRI. Secondary outcomes included its impact on other intracranial tumor volumes and hearing function.	Everolimus did not shrink VS enough to meet the primary endpoint, but it significantly slowed tumor growth in half of patients, and tumors sped up again when the drug was stopped. Re-starting treatment slowed growth once more. Other intracranial tumors didn’t shrink but showed slower growth on therapy. Hearing stayed stable during treatment [20].
Phase 2 Study of Bevacizumab in Children and Young Adults With NF 2 and Progressive Vestibular Schwannomas	NCT01767792	2013	Completed	*N* = 22	Single Group Assignment	This study evaluated bevacizumab for symptomatic VS in children and young adults with NF2, using high-dose induction followed by lower-dose maintenance in those with hearing stability or improvement. The primary outcome was hearing improvement at 24 weeks, and secondary outcomes included adverse events, treatment tolerability, durability of hearing response during maintenance, changes in pure-tone average, tinnitus-related distress, and durability of radiographic response.	About 41% of participants had a significant hearing improvement after 6 months of high-dose bevacizumab. All participants experienced some adverse events, but treatment was generally tolerated, with 95% completing induction without stopping due to side effects. Among those who improved initially, about 57% maintained hearing gains during low-dose maintenance therapy, and tumor-shrinkage responses of ≥ 20% were maintained in 71% of evaluable patients. Changes in pure-tone average were small, and tinnitus distress scores were modest [21].
Endostatin Study for Patients With Neurofibromatosis Type 2 (NF2) and *NF2*-Related Tumors (Endostatin)	NCT02104323	2014	Completed	*N* = 20	Single Group Assignment	This study evaluated the use of continuous intravenous recombinant human endostatin in NF2 patients with *NF2*-related tumors, including VSs, based on its ability to inhibit tumor blood vessel formation. The primary outcome was the change in tumor volume from baseline after each treatment course. Secondary outcomes included changes in hearing ability and changes in quality-of-life (QOL) scores after each course of therapy.	No results posted.
Study of RAD001 for Treatment of *NF2*-related Vestibular Schwannoma	NCT01345136	2015	Terminated	*N* = 4	Single Group Assignment	This study evaluated whether RAD001 could shrink or slow the growth of VS in NF2 patients. Participants received daily oral RAD001 for up to one year while tumor size and clinical status were monitored. The primary outcome was change in VS volume. Secondary outcomes included effects on hearing and the number of adverse events.	No results posted.
Icotinib Study for Patients With Neurofibromatosis Type 2 (NF2) and *NF2*-Related Tumors (Icotinib)	NCT02934256	2016	Completed	*N* = 10	Single Group Assignment	This study evaluated the preliminary effectiveness, safety, and tolerability of Icotinib Hydrochloride Tablets in patients with NF2, based on evidence that EGFR inhibition may slow VS growth and improve hearing. The primary outcome was the change in tumor volume from baseline after each treatment course, while secondary outcomes included changes in hearing ability and quality-of-life scores throughout treatment.	Among the 10 enrolled patients (mean age 23.8 years), one patient (10%) demonstrated an objective radiographic tumor response, with reductions of approximately 22–24% in bilateral VS. Of the seven evaluable patients, three (43%) achieved a hearing response. At 12 months, volumetric progression-free survival was estimated at 82%, and hearing progression-free survival at 69%. Treatment was generally well tolerated, with the most common mild to moderate adverse events being rash (90%), diarrhea (50%), myalgia (20%), and nausea or gastrointestinal discomfort (20%) [22].
Trial of Selumetinib in Patients With Neurofibromatosis Type II Related Tumors (SEL-TH-1601)	NCT03095248	2017	Terminated	*N* = 10	Parallel assignment with stratified cohorts (non-randomized)	This study evaluated selumetinib for *NF2*-related tumors in patients with tumor growth and/or hearing decline. It aimed to determine whether selumetinib could stop tumor growth, improve hearing over 24 weeks, enhance quality of life, and identify tumor markers relevant to treatment. The primary outcomes were hearing response at 24 weeks (improvement in word-recognition beyond the 95% critical threshold) and radiographic tumor response.	Only one patient was enrolled in Stratum 1 (VS cohort) for hearing assessment, and 0% achieved a hearing response at 24 weeks. For radiographic outcomes, 1/1 VS case and all 9 non-VS NF2 tumors were categorized as stable disease, with no radiographic responses observed in either group.
Study of Aspirin in Patients With Vestibular Schwannoma	NCT03079999	2018	Active, not recruiting	*N* = 97	Randomized controlled trial	This study is testing whether aspirin could slow VS growth and help preserve hearing. Participants will receive either aspirin or placebo until tumor progression, after which placebo patients could switch to open-label aspirin. The primary outcome is progression-free survival.	No results posted.
Innovative Trial for Understanding the Impact of Targeted Therapies in *NF2*-Related Schwannomatosis (INTUITT-NF2)	NCT04374305	2020	Active, not recruiting	*N* = 100	Parallel assignment within a platform/basket framework (randomized)	This multi-arm platform “basket” study tests multiple investigational therapies for *NF2*-related schwannomatosis, allowing patients with progressive VS, non-VS schwannomas, meningiomas, or ependymomas to receive different experimental drugs across sub-studies. Participants are screened and assigned to treatment arms, such as brigatinib or neratinib, and may switch to another substudy if their tumors progress. The primary outcome is radiographic response rate for each drug arm, and secondary outcomes assess safety and tolerability through treatment-emergent adverse events.	Interim results from 40 patients treated with brigatinib showed modest radiographic responses, with a 10% response rate in target tumors and higher activity across all tumors (23%), particularly in meningiomas and non-vestibular schwannomas, with no responses in target vestibular schwannomas. Tumor growth rates were reduced and 45% of patients had at least one responding tumor. Clinically, hearing improved in 35% of evaluable ears and pain decreased, with overall stable quality of life. Treatment was well tolerated with no grade 4 or 5 adverse events, representing promising but preliminary efficacy [23].
Phase 2 Clinical Trial of Crizotinib for Children and Adults With Neurofibromatosis Type 2 and Progressive Vestibular Schwannomas (NF110)	NCT04283669	2020	Completed	*N* = 12	Single Group Assignment	This study treats patients with NF2 and progressive VS using oral crizotinib, given continuously in 28-day cycles for up to 12 cycles or until progression or unacceptable toxicity. Tumor response is monitored by MRI every three cycles, and patients with progression stop treatment. Those who complete 12 cycles without progression may be eligible for retreatment if the tumor later regrows. The primary outcome is the volumetric response rate.	No results posted.
Nimodipine in Vestibular Schwannomas	NCT04801953	2021	Unknown status	*N* = 30	Randomized controlled trial	This randomized, double-blind study evaluates whether applying nimodipine directly to the facial and cochlear nerves during VS surgery can better preserve nerve function compared with placebo. Thirty patients undergoing microsurgical tumor resection will receive either local nimodipine or saline via a soaked gel-foam pad, with postoperative nerve outcomes assessed three months later. The primary outcomes are postoperative facial nerve function (House-Brackmann score) and hearing status (serviceable vs. non-serviceable on the Gardner-Robertson scale).	No results posted.
Study to Determine Optimal Dose, Evaluate the Efficacy and Safety of PRG-N-01 in Patients With Neurofibromatosis Type II	NCT07131722	2025	Not yet recruiting	*N* = 25	Dose-escalation, sequential assignment (randomized)	This study is testing Trineumin (PRG-N-01) in adults with *NF2*-related tumors to determine whether the drug is safe, tolerable, and capable of reducing tumor size or improving quality of life, including hearing. Participants take Trineumin daily for 96 weeks with regular clinic visits for monitoring. The study’s primary outcomes include identifying dose-limiting toxicities, determining the maximum tolerated dose and recommended Phase 2 dose, and assessing radiographic tumor responses through measures such as maximum tumor-size change rate, best overall response, objective response rate, duration of response, and progression-free survival. Secondary outcomes further evaluate tumor response durability, hearing function via pure-tone audiometry and word-recognition scores, changes in NF2-specific quality-of-life scores, overall adverse events, and exploratory blood-based biomarkers.	No results posted.

**Table 3:** Summary of phase 3 interventional clinical trials in vestibular schwannoma identified through ClinicalTrials.gov

Title	Trial ID	Study Start	Study Status	Enrollment	Interventional Model	Summary	Results
Corticosteroids in Prevention of Facial Palsy After Cranial Base Surgery	NCT00438087	2007	Completed	*N* = 313	Randomized controlled trial	This study evaluates whether high-dose corticosteroids given during and after surgery can reduce facial palsy in patients undergoing VS or other benign cranial-base tumor removal. Patients are randomized to receive either methylprednisolone or placebo, and facial nerve function is assessed before and after surgery to determine whether steroids lessen nerve dysfunction. The primary outcome is facial nerve function at 8 post surgery. The secondary outcome is facial nerve function on day 1 post surgery.	No results posted.
Stereotactic Radiation in Vestibular Schwannoma	NCT01449604	2011	Unknown status	*N* = 200	Randomized controlled trial	This study is designed to determine whether stereotactic radiosurgery (SRS) or stereotactic radiotherapy (SRT) is more effective for treating VS. Patients are randomized to receive either SRS or SRT, allowing direct comparison of the two approaches. The primary outcome is hearing status measured by audiogram, and secondary outcomes include the number of participants experiencing adverse events and changes in tumor size.	No results posted.
Steroids for Facial Nerve Function Protection in Post-surgical Vestibular Schwannoma Patients (SAF-NRVS)	NCT07116811	2025	Not yet recruiting	*N* = 364	Randomized controlled trial	This multicenter randomized, double-blind, placebo-controlled trial tests whether perioperative steroids improve facial nerve recovery after VS surgery in patients with good baseline and intraoperative nerve function. The primary outcome is the proportion of patients with good facial nerve function (House-Brackmann I-II) at 90 days. Secondary outcomes include early (10-day) and complete facial nerve recovery, Sunnybrook scores, and rates of adverse and serious adverse events within 10 and 90 days.	No results posted.

Most trials were completed (*n* = 9), with others active but not recruiting (*n* = 3), terminated (*n* = 3), not yet recruiting (*n* = 2), unknown (*n* = 2), actively recruiting (*n* = 1), or suspended (*n* = 1) (Fig. 2e-f). The limited number of active trials and presence of terminated/suspended studies highlight challenges in feasibility and development.

Most studies used single-group designs (*n* = 12), with fewer randomized controlled trials (*n* = 5) (Fig. 2g-h). Among the single-arm studies, common in Phase 2 (*n* = 10/15) and typically small (mean *n* = 12), only one incorporated external controls, while the rest lacked any comparative benchmark. In contrast, randomized trials were larger (mean *n* = 201) and more common in Phase 3 (*n* = 3/3). However, none of the randomized trials reported results, and the predominance of small, non-comparative, early-phase studies limits the strength of evidence.

Only 38% of trials (*n* = 8/21) had publicly available results, mostly from completed studies (*n* = 6/8). Notably, one-third of completed trials (3/9) did not report outcomes. Trials without results were not necessarily smaller, and reporting rates were lower in randomized trials (14%) than non-randomized studies (50%), partly due to ongoing status. Overall, limited reporting and transparency restrict access to high-quality evidence and hinder progress in the field.

### Clinical outcomes of investigated therapeutic agents

Therapeutic approaches and patient outcomes in VS remain heterogeneous, with no clear improvement in efficacy over time. Among completed trials, bevacizumab showed the most consistent activity, with meaningful hearing improvement and tumor control (NCT01207687, NCT01767792 [19]). Icotinib demonstrated modest activity, with limited radiographic response and some hearing improvement (NCT02934256 [21]), while lapatinib mainly achieved disease stabilization without tumor regression (NCT00973739 [22]). Everolimus slowed tumor growth in some patients but did not induce shrinkage, indicating limited benefit (NCT01490476 [20]). Selumetinib showed no significant hearing or radiographic responses, although interpretation is limited by the small sample size (NCT03095248). Intratympanic triamcinolone achieved reliable perilymph delivery but limited CSF penetration, reducing its therapeutic potential (NCT04658836 [18]). In an ongoing Phase 2 platform trial, brigatinib has shown modest activity, with some tumor growth reduction and hearing improvement (NCT04374305 [23]). Several trials lack reported results, and newer approaches, including gene therapy, remain under evaluation. Overall, the limited success of targeted therapies may reflect signaling pathway redundancy, molecular and clinical tumor heterogeneity, and adaptive activation of compensatory pathways. These mechanisms may allow VS cells to bypass single-pathway inhibition, contributing to modest, variable, or short-lived treatment responses. Future therapeutic strategies may require combination or multi-targeted approaches, guided by a better understanding of pathway redundancy, adaptive signaling responses, and the molecular profile of individual tumors.

### Emerging imaging biomarkers

Emerging studies are evaluating imaging biomarkers to improve patient stratification and mechanistic insight. One study (NCT05685836) uses ⁸⁹Zr-labeled bevacizumab PET/CT to assess tumor uptake and predict response to anti-VEGF therapy, while another (NCT07130851) employs functional MRI to map central vestibular pathway activity. Together, these approaches may help predict treatment response and characterize functional impairment, supporting more personalized, mechanism-based management and trial design.

## Epigenetics in vestibular schwannomas

### DNA methylation and epigenetic-phenotypic correlation

Early models suggested *NF2* loss could arise from promoter hypermethylation, but this is rare and not a major driver of VS [15]. Instead, genome-wide profiling reveals widespread methylation changes, particularly hypomethylation at developmental loci (e.g., *HOX* clusters), likely reactivating embryonic Schwann cell programs and promoting proliferation and impaired differentiation [11]. DNA methylation studies also define two epigenetic subgroups independent of *NF2* status: immune-enriched schwannomas (IES), with macrophage-rich, “immune hot” profiles and sensitivity to CSF1R inhibition, and immune-depleted schwannomas (IDS), with pro-angiogenic, VEGF-A⁺, “immune cold” profiles and relative resistance [12]. IES are typically diagnosed in adulthood and follow a favorable prognosis, whereas IDS present predominantly in childhood and are associated with poorer outcomes, demonstrating an epigenetic-phenotypic correlation [12].

Methylation profiling (Illumina 450 K) further identifies VS as a distinct, homogeneous subgroup across sporadic and hereditary cases, contrasting with the heterogeneity of other schwannomas and suggesting a unique baseline methylation profile of vestibulocochlear nerve Schwann cells [47].

### Chromatin remodeling and histone modifiers

VS cells depend on chromatin-modifying enzymes for survival. Histone deacetylases (HDACs) compact chromatin and repress gene expression; their inhibition (e.g., AR42) restores tumor-suppressive and pro-apoptotic genes, including negative regulators of PI3K/AKT, promoting cell death [13]. The dual inhibitor CUDC907 targets both PI3K signaling and HDAC activity, and notably also reduces YAP levels in primary VS cells, suggesting convergent suppression of multiple merlin-dependent vulnerabilities [48, 49].

These findings highlight a common principle: combining HDAC inhibition with genetic signaling blockade enhances antitumor effects in preclinical studies compared to single agents, positioning HDACs as a key therapeutic vulnerability. Additionally, inhibition of *SIRT2*, a class III HDAC, induces selective necrotic death in merlin-deficient Schwann cells, suggesting a synthetic-lethal strategy [50].

### Non-coding RNAs

Epigenetic reprogramming in VS involves deregulated non-coding RNAs (ncRNAs). Global profiling shows widespread microRNA dysregulation regardless of *NF2* status, including upregulation of the oncogenic 14q32 miRNA cluster [51]. Some miRNAs act as tumor suppressors, such as miR-205, which inhibits proliferation, and miR-1, which promotes apoptosis [52]. Disruption of miRNA-mRNA networks enhances ErbB signaling and weakens cell-cycle control, promoting tumor progression [53]. Long non-coding RNAs also contribute, with BRCAT54 implicated in proliferation and survival [54].

ncRNAs further influence the tumor microenvironment. Tumor-secreted miR-431, found in extracellular vesicles, may act paracrinally to modulate immune and stromal cells, promoting growth and immune evasion [55]. Clinically, ncRNA profiles show biomarker potential: miR-449a/b, miR-15/16 − 1, and hypoxamiRs are associated with non-serviceable hearing, while rapid tumor growth correlates with upregulated miR-29abc, miR-19, miR-340-5p, miR-21, miR-221, and downregulated miR-744 and let-7b. These findings support ncRNA signatures as potential prognostic biomarkers and tools for patient stratification [56].

### SOX10 regulation

Loss of lineage regulators intersects with epigenetic mechanisms in shaping merlin-null VS identity. *SOX10*, a master regulator of Schwann cell development, is reduced at both the protein and mRNA level in merlin-null schwannoma cells. This loss impairs induction of the downstream transcription factors *KROX20* and *OCT6* and prevents nuclear localization of NFATC4, collectively disabling the transcriptional program required for terminal Schwann cell differentiation and quiescence. *SOX10*-deficient cells instead acquire increased proliferation, enhanced focal adhesions, and upregulated PDGFRβ expression, features characteristic of VS. In vitro reintroduction of *SOX10* into merlin-null VS cells restores *KROX20* and myelin gene induction, suppresses PDGFRβ, relocalizes NFATC4 to the nucleus, and reduces proliferation, identifying the *SOX10* pathway as a central and potentially reversible mechanism underlying the merlin-null schwannoma state [14]. PDGFRβ has also been targeted preclinically with the RTK inhibitors imatinib and nilotinib, both of which inhibit VS cell proliferation in vitro, though no clinical data yet exist [57, 58].

## Molecular mechanisms underlying hearing loss in vestibular schwannoma

### Mechanisms of hearing loss in VS

Hearing loss is a major determinant of quality of life in VS. Although initially attributed to cochlear nerve compression, evidence supports multiple mechanisms. VS causes greater hearing and vestibular dysfunction than cerebellopontine angle meningiomas of similar size and location, suggesting factors beyond compression [59].

Pathologic studies show ipsilateral hair cell loss, endolymphatic hydrops, and proteinaceous precipitates compared with the contralateral ear [60]. Perilymph analyses also identify inflammatory markers linked to worse hearing [61]. Supporting fluid dysregulation, anti-VEGF therapy can improve hearing rapidly, sometimes before tumor shrinkage [41]. Tumor-secreted and inflammatory factors also contribute: VS secretions induce TNF-α-mediated cochlear damage in vitro [62], *CXCR4* expression correlates with greater hearing loss [63], and angiotensin receptor blockers may preserve hearing via anti-inflammatory effects [64].

### VS genetics and hearing loss

Genotype-phenotype correlations may help explain variability in hearing outcomes in VS. Truncating NF2 mutations are associated with earlier age of onset of symptoms and earlier age of hearing loss, but the rate of hearing decline once it begins is not clearly different across mutation types [18, 65]. Whole-genome expression profiling of VS tissue has identified differential expression of *PEX5L*, *RAD54B*, *PSMAL*, and *CEA* between tumors associated with good versus poor hearing [66]. At the cochlear level, *NF2*-driven vascular permeability leads to intra-labyrinthine protein accumulation and cochlear aperture obstruction, changes strongly associated with hearing loss that can occur independently of tumor size [67]. These findings suggest that *NF2* mutation type influences the timing of hearing loss onset but that downstream gene expression differences within VS tumors may better account for the variability in hearing outcomes among individual patients.

### VS epigenetics and hearing loss

Epigenetic-phenotypic mechanisms also contribute to variations in hearing. *TP73* methylation has been associated with hearing loss, with affected patients showing worse pure tone averages and speech discrimination [68]. The *TP73*-encoded transcription factor p73 regulates neuronal apoptosis and may influence radiosensitivity [69]. However, the role of epigenetics in vestibular dysfunction remains largely unexplored.

## Limitations

This study was conducted as a hybrid narrative and structured review rather than a fully systematic review or meta-analysis. A systematic search strategy and predefined selection framework were specifically applied to the ClinicalTrials.gov component to enable a rigorous and reproducible assessment of prospective interventional studies and facilitate comparison of therapeutic outcomes across clinical trials. In contrast, the broader literature synthesis was intentionally narrative to allow integration of heterogeneous evidence spanning preclinical, molecular, genetic, epigenetic, and translational domains that would be difficult to capture within a narrowly defined systematic framework. Nevertheless, because the narrative component was not conducted according to a preregistered protocol and did not include formal risk-of-bias assessment or quantitative meta-analysis, study selection and interpretation may be subject to bias, and some relevant studies may not have been identified or included.

The proposed mechanistic framework for VS, including Hippo/YAP-TAZ, PI3K/AKT/mTOR, VEGF, MAPK, adhesion/motility pathways, and epigenetic regulation, is primarily derived from preclinical models, small surgical or molecular series, and pathway-focused studies. These may not fully reflect the diversity of sporadic, *NF2*-related, and schwannomatosis-associated tumors. Translational findings may be influenced by model systems, publication bias, and limited sampling of treatment-naïve human tissue, with many lacking independent validation. Similarly, the epigenetic and ncRNA landscape is based on small, methodologically diverse cohorts, making proposed subgroups and signatures provisional. The use of *NF2*-associated schwannomas as a model for sporadic VS is also a limitation, as sporadic tumors may exhibit distinct molecular and epigenetic heterogeneity that is not fully captured by *NF2*-related disease models, potentially leading to overly generalized mechanistic interpretations.

In addition, Phase 4 and observational studies were not systematically included in the therapeutic synthesis. As a result, real-world treatment experience, including longer-term effectiveness, safety, and durability of response with agents such as bevacizumab, may be incompletely represented, although such evidence remains important given the limited number of completed prospective VS trials. This review focused on the genetic and epigenetic landscape of VS and their implications for targeted therapies; however, the tumor immune microenvironment represents another critical and rapidly evolving area that warrants dedicated discussion, particularly given emerging interest in immunotherapeutic strategies such as CSF1R inhibition. Moreover, figures are schematic and conceptual rather than quantitative. Finally, cost, access, long-term quality-of-life outcomes, and comparisons with other skull-base tumors were not systematically assessed.

## Future directions and conclusion

Future directions in VS research include: (1) integrating multi-omic profiling (genome, methylome, transcriptome) into routine evaluation to classify tumors by pathway dependencies and immune/angiogenic states (IES vs. IDS); (2) conducting biomarker-stratified clinical trials with standardized volumetric measures and co-primary, patient-relevant functional endpoints; and (3) validating combinatorial signaling and epigenetic therapies to identify synergistic treatment strategies.

Clinically, success should be defined by durable tumor control with maximal preservation of patient-relevant outcomes such as hearing and facial nerve function. Advances in biological insight, therapeutic precision, clinical trial design, and patient-centered care are expected to shift VS management from invasive default approaches toward personalized, mechanism-based strategies that prioritize quality of life.

## Supplementary Information

Below is the link to the electronic supplementary material.

Supplementary Material 1

## Data Availability

No datasets were generated or analysed during the current study.
