# Supplementary material for: Vestibular schwannoma: genetic and epigenetic mechanisms, hearing loss, and emerging therapies
Source: J Neurooncol. 2026 May 16;178(1):3. doi: 10.1007/s11060-026-05621-4 (PMC13179888; doi:10.1007/s11060-026-05621-4)
Supplement: Supplementary file 1 — Supplementary Material 1 [file 11060_2026_5621_MOESM1_ESM.docx]

**Identification of studies via ClinicalTrials.gov**

Trials removed *before screening*:

Trials marked as observational (n = 24)

Trials marked as Early Phase 1 (n = 3)

Trials marked as Phase 4 (n = 2)

Trials identified from ClinicalTrials.gov (n = 92)

**Identification**

Trials excluded

Trials not specified as Phase 1, 2, or 3 (n = 35)

(n = )

Trials screened

(n = 63)

**Screening**

Trials excluded:

Trials not relevant to vestibular schwannoma (n = 2)

Non-therapeutic trials (n = 5)

Trials assessed for eligibility

(n = 28)

Trials included in review

(n = 21)

**Included**
